# Hyperlipasemia Sans Pancreatitis: A Case Series

**DOI:** 10.7759/cureus.47781

**Published:** 2023-10-27

**Authors:** Jisa George, Kothai Gnanamoorthy, Prasanna Karthik Suthakaran, Krishna Venkatesh Baliga

**Affiliations:** 1 Internal Medicine, Employees State Insurance Corporation (ESIC) Medical College & Post-Graduate Institute of Medical Sciences and Research (PGIMSR), Chennai, IND; 2 Internal Medicine, SRM Medical College, Kattankulathur, IND; 3 Internal Medicine, Saveetha Medical College Hospital, Saveetha Institute of Medical And Technical Sciences (SIMATS), Kanchipuram, IND; 4 Nephrology, Employees State Insurance Corporation (ESIC) Medical College & Post-Graduate Institute of Medical Sciences and Research (PGIMSR), Chennai, IND

**Keywords:** organophosphate poisoning, sle, crohn's disease, chronic kidney disease, pancreatitis, hyperlipasemia

## Abstract

Acute pancreatitis results in inflammation and autodigestion of pancreatic acinar cells leading to the elevation of pancreatic enzymes, namely, amylase and lipase. Serum lipase levels have long been considered a hallmark of acute pancreatitis. However, pancreatitis is not always the cause of elevated serum lipase levels. This series presents four patients who had elevated serum lipase levels without any demonstrable damage to the pancreas on imaging. On further evaluation, one of the patients was found to have acute on chronic kidney disease (CKD) whose lipase levels settled later. A patient presenting with an episode of acute gastroenteritis, later diagnosed to have Crohn’s disease, also had hyperlipasemia, which improved after a course of initial antibiotics. Non-gastrointestinal causes, such as lupus nephritis and organophosphate (OP) poisoning, also had elevated lipase levels on presentation, in which the hyperlipasemia settled with supportive treatments. It is important to remember other causes of elevated lipase levels in patients with a normal pancreas on imaging studies.

## Introduction

Acute pancreatitis is diagnosed by the presence of two of the following: 1) typical abdominal pain, 2) characteristic findings on computed tomography (CT) of the abdomen and/or 3) greater than three times the reference upper normal limit for serum amylase and/or lipase levels [1]. Serum lipase levels have been shown to be a more specific biomarker of acute pancreatitis [2]. Apart from pancreatic acinar cells, lipase is also found elsewhere in the gastrointestinal tract, including the esophagus, duodenum, stomach and colon [3]. It has also been observed that mild elevations in serum lipase levels are seen in peptic ulcer disease and other hepatobiliary diseases [4]. Any patient presenting with abdominal pain and elevated lipase levels prompts the clinician to look for pancreatic causes of the abdominal pain. However, the literature suggests that there can be alternative and non-pancreatic causes of abnormal and significant elevation of serum lipase levels [5]. It is important for the treating physician to look for these alternative causes to avoid erroneously diagnosing hyperlipesemia as related only to pancreatitis. This will alert the clinician to avoid overemphasis on lab reports and improve the patient diagnosis and outcome. Here, we report a case series of patients who presented with elevated lipase levels of different aetiologies other than pancreatitis.

## Case presentation

Patient 1

A 57-year-old lady presented with abdominal pain, nausea, vomiting and abdominal distension of three days' duration and loose stools for a day. There was no history of decreased urine or blood in urine. She had no other known comorbidities. On examination, she was tachypnoeic but haemodynamically stable with bi-basal crepitations. The abdomen was soft with mild right iliac fossa tenderness. Her baseline investigations revealed normocytic normochromic anaemia, neutrophilic leucocytosis, elevated renal parameters (urea 137 mg/dl and creatinine 6.9 mg/dl), hypocalcaemia (6.95 mg/dl) and hyperphosphatemia (7.5 mg/dl). Arterial blood gas analysis revealed high anion gap metabolic acidosis with compensated respiratory alkalosis. The baseline characteristics of the patients are given in Table 1.

**Table 1:** Baseline characteristics of the patients WBC: white blood cell, SGOT: serum glutamic-oxaloacetic transaminase, SGPT: serum glutamic-pyruvic transaminase

	Patient 1	Patient 2	Patient 3	Patient 4
Age (in years)	57	56	34	58
Sex	Female	Female	Female	Male
Haemoglobin (g/dL)	9.5	9.2	10.4	12.1
Total WBC count (cells/µL)	18,200	6,500	4,200	13,500
Blood urea (mg/dL)	157.8	46	37.3	35
Serum creatinine (mg/dL)	7.24	1.8	1.05	0.8
Serum sodium (mEq/l)	120	138	130	144
Serum potassium (mEq/l)	4.04	3.9	3.7	3.8
Total bilirubin (mg/dL)	0.9	0.8	1.2	2.1
SGOT (U/L)	24	26	18	73
SGPT (U/L)	27	32	27	42

An initial diagnosis of acute on chronic kidney disease (CKD) due to sepsis was made. As the patient had persistent epigastric pain, serum lipase levels were evaluated to rule out acute pancreatitis. The serum lipase was markedly elevated (4586 IU/L, N: up to 43 IU/L). The ultrasound abdomen showed normal-sized kidneys. Non-contrast CT of the abdomen and pelvis showed mild jejunal dilatation and no evidence of pancreatitis. She was treated conservatively with intravenous cefotaxime 1 g twice daily and metronidazole 500 mg thrice daily and other supportive measures. The patient showed improvement in her abdominal pain. Her serial lipase levels showed a falling trend after day 5. Her serial renal parameters showed gradual recovery, and she was on conservative management till the time of discharge after 10 days.

Patient 2

A 56-year-old female, with known hypothyroid, on stable levothyroxine replacement (100 μg/day), presented with complaints of fever, diarrhea and vomiting of three days' duration. There were no other significant symptoms.

**Figure 1:**
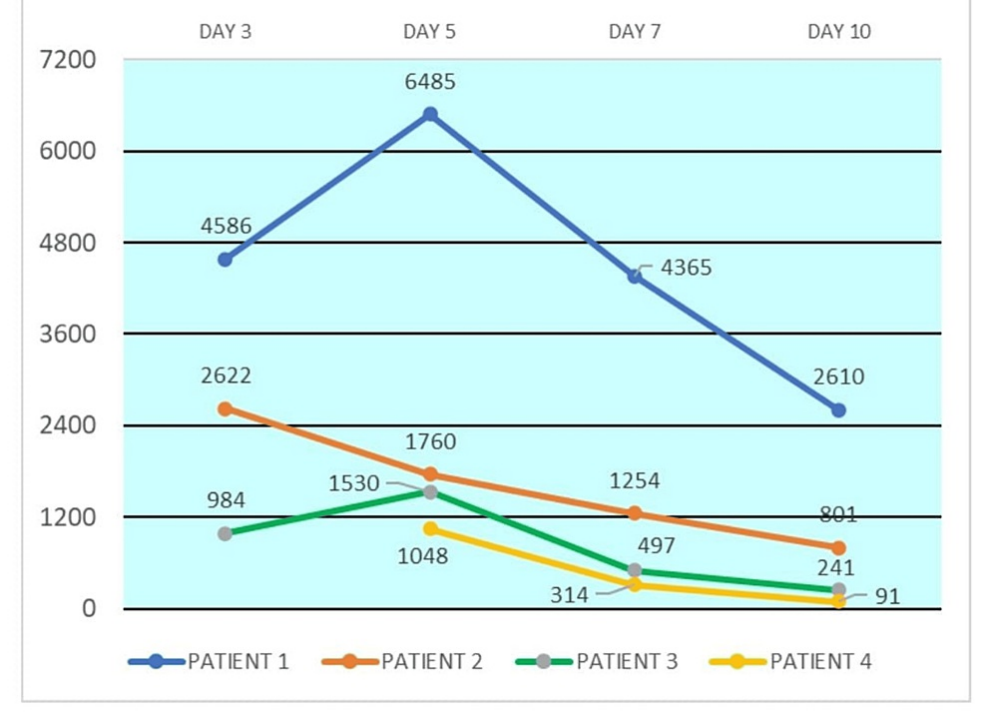
Temporal trends of the serum lipase levels (IU/L)

On examination, she was dehydrated and had mild epigastric tenderness. An initial diagnosis of acute gastroenteritis was made. Her initial baseline investigations were within normal limits. Stool examination was unremarkable. As the patient had persistent loose stools and epigastric pain, serum lipase was done to rule out pancreatitis. It was markedly elevated (2,622 IU/L, N: up to 43 IU/L). Contrast-enhanced CT of the abdomen revealed circumferential mucosal thickening involving the terminal ileum and ileocecal junction along with diffuse peritoneal thickening involving the entire abdominal cavity. A possibility of ileocecal tuberculosis (TB) or Crohn’s disease was considered. She underwent colonoscopy-guided biopsy, which revealed diffuse interstitial inflammatory infiltrate suggestive of Crohn’s disease. She was started on oral steroids (prednisolone 30 mg per day tapered to 15 mg over two weeks) and mesalamine enema to which the patient responded well. Serial lipase levels also showed a decreasing trend (Figure 1). 

Patient 3

A 34-year-old lady presented with progressive anasarca, diffuse abdominal pain and frequent vomiting of three months' duration. She had no decreased urine output or bloody urine. She had a known hypothyroidism and was on irregular levothyroxine replacement (75 μg/day).

On examination, she had periorbital puffiness and bilateral pitting pedal edema and was tachypnoeic at rest. She had bilateral basal crepitations and diffuse abdominal tenderness. Her initial investigations revealed bicytopenia, elevated erythrocyte sedimentation rate (ESR) of 83 mm for the first hour and thyroid-stimulating hormone (TSH) of 11.8 μIU/L. As the patient had loose stools and persistent abdominal pain, serum lipase was evaluated to rule out pancreatitis. The serum lipase was elevated (984 IU/L, N: up to 43 IU/L). Non-contrast CT of the abdomen revealed diffuse circumferential wall thickening of the pyloric canal, distal ileum, cecum and ascending and transverse colon with prominent mesenteric, retroperitoneal and bilateral iliac lymph nodes suggestive of inflammatory colitis and mild ascites with a normal pancreas. Her diarrhoea and abdominal pain resolved with supportive treatment. She developed progressively worsening dyspnoea from day 5. She was found to have a right-sided moderate pleural effusion, which was tapped and analysed. It was found to be exudative. As she had subnephrotic-range proteinuria with polyserositis and bicytopenia, she underwent an autoimmune workup. Her anti-nuclear antigen (ANA) was strongly positive (3+) with low complement (C3, C4) levels. Renal biopsy showed lupus nephritis stage 4. 

A diagnosis of systemic lupus erythematosus (SLE) with lupus nephritis in acute flare was made, and she was started on pulse steroid therapy (three daily pulses of intravenous methylprednisolone 750 mg) along with pulse cyclophosphamide (0.75 g/m^2^) to which the patient responded well. Serial lipase levels also showed a decreasing trend. She was discharged after 10 days on oral corticosteroids and followed up with the nephrology clinic for monthly cyclophosphamide pulse therapy. 

Patient 4

**Figure 2:**
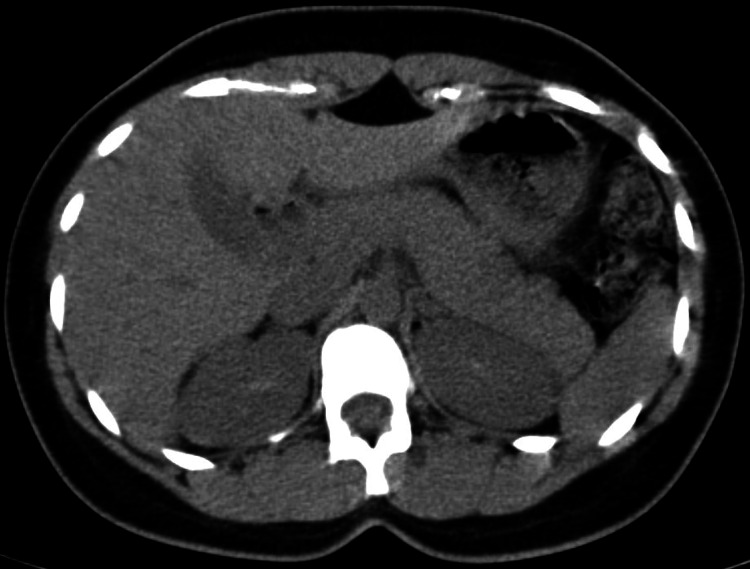
CT abdomen of patient 4, showing a normal pancreas

A 58-year-old male was admitted with a history of consumption of around 10 ml of organophosphate liquid under the influence of alcohol. He was a known hypertensive and diabetic, on irregular medication. On arrival to the hospital, he was drowsy, his blood pressure was 220/120 mm Hg, and pulse rate was 102 beats/minute. He was also tachypnoeic with bilateral extensive lung crepitations. Pupils were bilateral constricted. He was started on atropine loading dose (a total of 8 mg initially given as 2 mg over five-minute interval till atropinisation was achieved), followed by continuous infusion (rate 1.6 mg per hour). Intravenous nitroglycerine infusion was also started. The patient showed a mild improvement in his vitals, pupils dilated to 3 mm and blood pressure settled to normal range within 12 hours of admission. On the third day, he was intubated and put on mechanical ventilation due to sudden desaturation and persistent respiratory distress. Baseline investigations revealed mild leucocytosis, normal renal parameters and hypokalaemia (2.1 meq/L), mild transaminitis (serum glutamic-oxaloacetic transaminase (SGOT): 506/serum glutamic-pyruvic transaminase (SGPT): 154 IU/L). Serum lipase levels were evaluated to rule out any co-existent ethanol-related pancreatitis. It was elevated to 1048 IU/L (N: up to 43 IU/L). Non-contrast enhanced CT of the abdomen done on day 5 showed a normal pancreas (Figure 2). Gradually, he improved and was weaned off the ventilator on day 14. His serum lipase showed normalisation by day 10. He was discharged on day 20.

## Discussion

Lipase is a good serological test for the diagnosis of pancreatitis. The sensitivity and specificity of serum lipase for pancreatitis at a cut-off of three times the upper limit of normal ranges from 64% to 100% and from 99% to 100%, respectively [6].

The first patient in this case series was diagnosed to have acute on CKD. Patients with renal dysfunction can display a small or moderate increase in serum pancreatic enzymes. Twenty percent of pancreatic enzymes are excreted by the kidney; hence, in patients with CKD, pancreatic enzymes have been found to be elevated. Such an increase has been attributed to a reduced glomerular filtration rate or to subclinical pancreatic damage [7]. Manju et al. showed that there is a significant increase in pancreatic amylase and lipase levels in patients with CKD with or without end-stage renal disease (ESRD), which was also in corroboration with the previous findings by Jiang et al. [8,9]. Other hypothetical mechanisms include regurgitation of pancreatic enzymes from ductal obstruction for precipitation of inspissated secretion, electrolyte disturbances and mainly altered calcium phosphorus homeostasis [10]. It might be worthwhile to consider a different reference range for amylase and lipase levels in CKD to avoid erroneously diagnosing pancreatitis.

The second patient was found to have Crohn’s disease with hyperlipasemia. The postulated reason for such an increase of serum lipase in inflammatory bowel disease (IBD) patients is an extra-pancreatic release of these enzymes from the inflamed bowel and also intestinal reabsorption of the released lipase [11]. However, pancreatitis can be a rare extraintestinal manifestation of IBD, secondary to duodenal fistulas, ampullary or primary pancreatic Crohn's disease, gallstones, primary sclerosing cholangitis or even drugs used in treatment, such as azathioprine [12]. In clinical practice, the symptoms of pancreatitis may sometimes be difficult to distinguish from active IBD with diarrhoea and/or abdominal pain. 

The third patient in the case series was diagnosed with SLE and lupus nephritis. Recent studies support the role of SLE as the primary etiologic factor of pancreatitis with drug toxicity having some role [13]. The mechanisms involved are postulated to be vasculitis, microthrombi and intimal thickening. Hypothetical mechanisms include complement activation, hypotension and autoimmune reactions secondary to anti-pancreatic antibodies. It can occur both during a generalized flare and during disease quiescence, although the latter seems to be more common [14]. In our patient, renal dysfunction and SLE both could have contributed to the elevated lipase levels. Goyal et al. reported a case of SLE with ESRD with pancreatic panniculitis resulting in elevated pancreatic enzymes [15]. Hasselbacher et al. reported an increase in the levels of amylase and lipase in SLE patients without any abdominal cause or renal dysfunction [16].

The fourth patient in the case series developed hyperlipasemia following OP poisoning. It has been associated with hyperlipasemia due to the fact that acute pancreatitis is caused by excessive cholinergic stimulation of the pancreas by the OP compounds [17]. In ICU patients with critical illness/multi-organ failure, pancreatic hypoperfusion with cellular stress may also be responsible for lipase elevations. Moreover, pancreatic enzymes in the gut may enter the submucosa and subsequently the systemic circulation during times of gut ischaemia from the reduced splanchnic flow [18]. In a retrospective study by Lee et al., it was observed that hyperamylasaemia associated with hyperlipasemia was seen in patients following OP poisoning, indicating a silent acute pancreatitis [19]. Similarly, Sahin et al. showed that 12.6% of patients admitted with OP poisoning developed acute pancreatitis, with both elevated serum amylase and lipase levels [20].

## Conclusions

Serum lipase levels have been used to diagnose acute pancreatitis in patients presenting with upper abdominal pain. However, we have to keep in mind the other causes of the elevation of serum lipase in the absence of pancreatitis. We have highlighted a series of four patients who have presented to us with a variety of clinical manifestations and were found to have elevated serum lipase levels. The imaging of the pancreas showed a structurally normal looking pancreas, which lead to the further evaluation and identification of other causes of hyperlipasemia. Clinicians should be well aware of the non- pancreatic aetiology of hyperlipasemia and its possible mechanisms to avoid misdiagnosis and overemphasis on altered biochemical lab parameters.
